# Complete mitochondrial genome of Mekong fighting fish, *Betta smaragdina* (Teleostei: Osphronemidae)

**DOI:** 10.1080/23802359.2021.1882893

**Published:** 2021-03-11

**Authors:** Nararat Laopichienpong, Syed Farhan Ahmad, Worapong Singchat, Aorarat Suntronpong, Tavun Pongsanarm, Kornsuang Jangtarwan, Jakaphan Bulan, Tanawat Pansrikaew, Thitipong Panthum, Nattakan Ariyaraphong, Navapong Subpayakom, Sahabhop Dokkaew, Narongrit Muangmai, Prateep Duengkae, Kornsorn Srikulnath

**Affiliations:** a Laboratory of Animal Cytogenetics and Comparative Genomics (ACCG), Department of Genetics, Faculty of Science, Kasetsart University, Bangkok, Thailand; b Special Research Unit for Wildlife Genomics (SRUWG), Department of Forest Biology, Faculty of Forestry, Kasetsart University, Bangkok, Thailand; c Department of Aquaculture, Faculty of Fisheries, Kasetsart University, Bangkok, Thailand; d Department of Fishery Biology, Faculty of Fisheries, Kasetsart University, Bangkok, Thailand; e Center for Advanced Studies in Tropical Natural Resources, National Research University-Kasetsart University (CASTNAR, NRU-KU), Kasetsart University, Bangkok, Thailand; f Center of Excellence on Agricultural Biotechnology (AG-BIO/PERDO-CHE), Bangkok, Thailand; g Amphibian Research Center, Hiroshima University, Higashihiroshima, Japan

**Keywords:** Fighting fish, mitogenome, bioresource, bubble-nesting group

## Abstract

Mekong fighting fish (*Betta smaragdina*) are found in Northeast Thailand. A complete mitochondrial genome (mitogenome) of *B. smaragdina* was assembled and annotated. Mitogenome sequences were 16,372 bp in length, with slight AT bias (59.8%), containing 37 genes with identical order to most teleost mitogenomes. Phylogenetic analysis of *B. smaragdina* showed closer relationship with *B. splendens and B. mahachaiensis* as the bubble-nesting group, compared to the mouthbrooder group (*B. apollon*, *B. simplex*, and *B. pi*). Results will allow the creation of a reference annotated genome that can be utilized to sustain biodiversity and eco-management of betta bioresources to improve conservation programs.

Southeast Asia, including the mainland and numerous islands, has a hot and humid climate that supports high biodiversity and bioresources. Fighting fish or betta (*Betta* spp.) were studied for their fighting and ornamental attributes. Long-term artificial crossbreeding among different species has honed their aggressive behavior and body features, resulting in many novel inbred lines (Witte and Schmidt 1992; Ramos and Gonçalves 2019). However, artificial selection has resulted in inbreeding and outbreeding depression between different betta species. The biodiversity of betta is being lost more rapidly now than at any time in the past several million years, with the invasion of hybrids introduced into the wild leading to genetic admixture (Beer et al. 2019). This is a very serious problem in the context of local conservation and indigenous species/lines. Genetic profiles for each local species must be resolved as a matter of urgency. One such species is the bubble-nesting Mekong fighting fish (*Betta smaragdina*) which is found in Northeast Thailand. However, the lack of a reference mitochondrial genome (mitogenome) has, to date, limited understanding of the genetic basis of aggression in this species. A sample of *B. smaragdina* was collected from Dong Luang, Mukdahan Province, Thailand (16.7966°N, 104.6238°E) and then stored in the Thailand Natural History Museum (no. THM21222). Whole genomic DNA was extracted in accordance with the standard salting-out protocol (Supikamolseni et al. 2015), and next-generation sequencing was performed using an Illumina HiSeq platform at Vishuo Biomedical (Thailand) Ltd. (Bangkok, Thailand). The quality of Illumina reads was evaluated with FastQC and the raw reads were trimmed to discard adapters using Trimmomatic software (Bolger et al. 2014). The filtered Illumina paired end reads were then assembled using a mitochondrial genome toolkit, MitoZ version_2.4-alpha (Meng et al. 2019) and processed with multiple modules including raw data pretreatment, de novo assembly, candidate mitochondrial sequences searching, and mitogenome annotation. A phylogenetic tree was constructed using Bayesian inference with MrBayes version 3.2.6 (Huelsenbeck and Ronquist 2001). The Markov chain Monte Carlo process was used to run four chains simultaneously for 1 million generations. After the log-likelihood value plateaued, a sampling procedure was performed every 100 generations to obtain 10,000 trees, and a majority-rule consensus tree with average branch lengths was provided.

A total of 118,164 individual reads gave an average coverage of around 170X for the generated contigs. Complete mitogenome sequences consisted of 16,372 bp (GenBank accession number: MW292561, SRA: SRR12599606, BioProject: PRJNA661175), containing 37 genes and a control region (CR), which indicated a conserved pattern of mitochondrial genome architecture in Betta species. Bioinformatic analysis of whole mtgenome comparisons for a total of 54 teleost species revealed a similar pattern of gene arrangement (Miya et al. 2013). Overall AT content for the mitogenome was 59.8%. Average nucleotide diversity among seven *Betta* mitogenomes was determined at 17.493 ± 2.453%. Four conserved blocks including CSB-D, CSB1, CSB2, and CSB3 were characterized in *B. smaragdina*. Previous findings suggest that these blocks within the CR region remain conserved across the mtgenomes of teleost lineages (Lee and Kocher 1995; Prakhongcheep et al. 2017; Ponjarat et al. 2019; Ahmad et al. 2020; Singchat et al. 2020). We annotated the mitogenome for repeats identification using RepeatMasker, version-4.1.1 (Smit et al. 2015) and found a total of six repeat elements. Tandem repeats have also been found previously in the mtgenomes of several *Betta* species including *B. pi* (AB920288) and *B. splendens* (AB571120 and KR527219) (Song et al. 2016; Prakhongcheep et al. 2017; Ponjarat et al. 2019; Ahmad et al. 2020; Singchat et al. 2020), suggesting that the CR had large variation in different fighting fish species. Twenty-two teleosts including seven betta mitogenomes were compared using CLUSTALW based on 12 concatenated protein-coding genes without *ND6*, and a phylogenetic tree was constructed using Bayesian inference with MrBayes version 3.2.6 (Huelsenbeck and Ronquist 2001). The close group comprising *B. splendens*, *B. mahachaiensis*, and *B. smaragdina* formed a monophyletic clade, consistent with Sriwattanarothai et al. (2010) as the bubble-nesting group, whereas the three remaining bettas (*B. apollon*, *B. simplex*, and *B. pi*) were classified to the mouthbrooder group (Ruber et al. 2004) (Figure 1).

**Figure 1.**
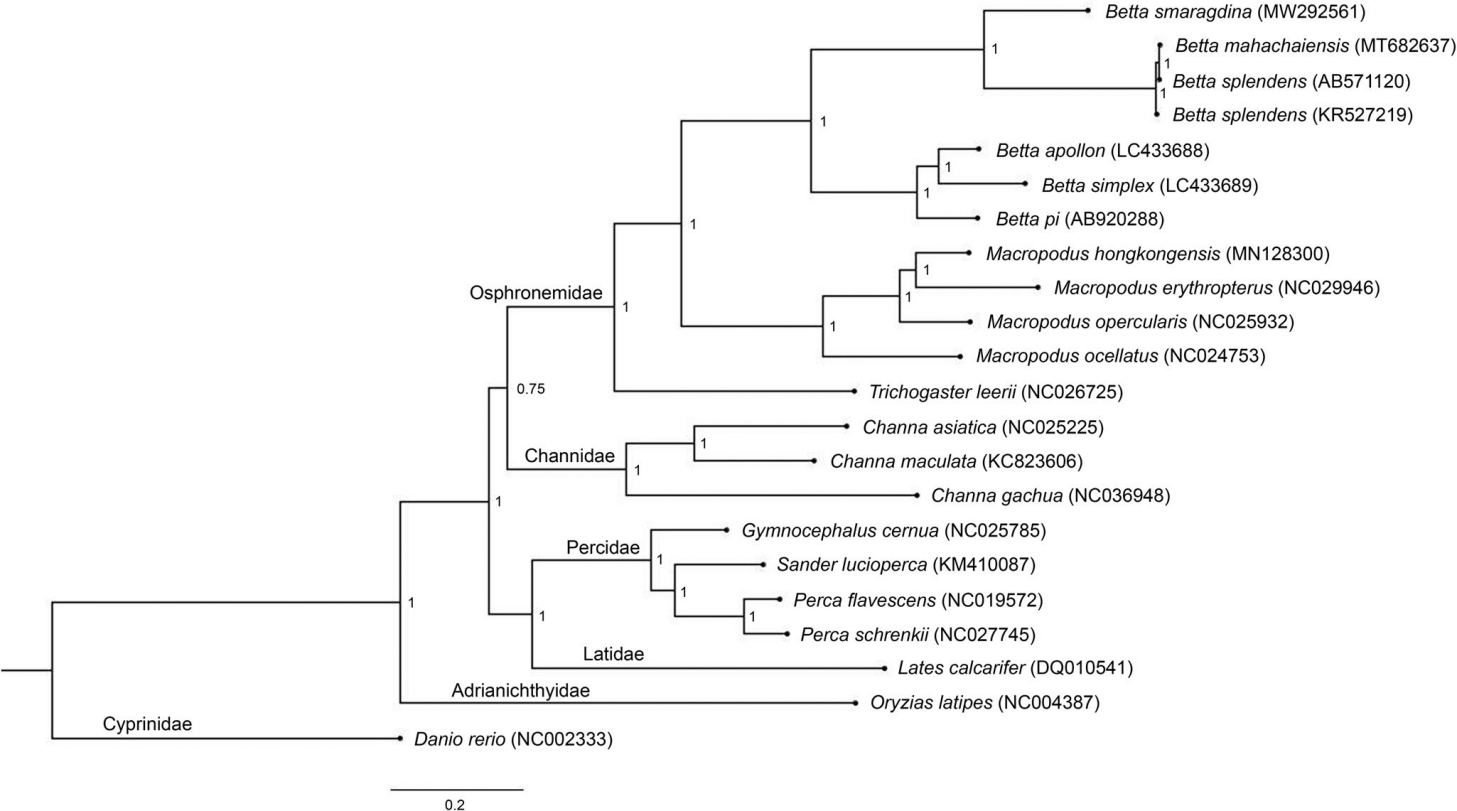
Phylogenetic relationships among 12 concatenated mitochondrial protein-coding genes, without *ND6* sequences of 22 mitochondrial genomes, including *Danio rerio* as the outgroup using Bayesian inference analysis. The complete mitochondrial genome sequence was downloaded from GenBank. Accession numbers are indicated in parentheses after the scientific names of each species. Support values at each node are Bayesian posterior probabilities, while branch lengths represent the number of nucleotide substitutions per site.

## Data Availability

Data supporting the study findings are available in GenBank through the NCBI at https://www.ncbi.nlm.nih.gov. Isolated mitogenome reads were deposited at NCBI SRA database (accession ID: SRR12599606), and the assembled mitogenome sequences are available in GenBank (accession ID: MW292561).
